# The impact of physical exercise on death anxiety in older adults: the chain-mediating role of self-efficacy and psychological resilience

**DOI:** 10.3389/fpsyt.2025.1608028

**Published:** 2025-11-20

**Authors:** Feixuan Li, Jiahui Peng, Deqiao Zhou

**Affiliations:** 1 College of Physical Education and Sport Science, Qufu Normal University, Jining, China; 2 School of Physical Education, Shandong University, Jinan, China

**Keywords:** physical exercise, older adults, death anxiety, self-efficacy, psychological resilience, chain mediation

## Abstract

**Background:**

With the rapid global aging trend, understanding death anxiety among the elderly has become a key focus in mental health research. Grounded in a biopsychosocial perspective, this study aims to examine the association between physical exercise and death anxiety in older adults and to explore the potential mediating roles of self-efficacy and psychological resilience within a cross-sectional framework.

**Methods:**

Using a cross-sectional design, the study surveyed 772 Chinese individuals aged 60 and above. Data were collected via the Physical Activity Rating Scale, Death Anxiety Scale, General Self-Efficacy Scale, and Psychological Resilience Scale. Correlation analysis and multiple regression were conducted using SPSS 27.0, and the hypothesized mediating effects were tested using the Bootstrap method in PROCESS 4.0.

**Results:**

Physical exercise was significantly negatively correlated with death anxiety (r = -0.740, p < 0.01) and was a significant negative correlate in the regression model (β = -0.196, p < 0.001). Self-efficacy (r = -0.827, p < 0.01) and psychological resilience (r = -0.854, p < 0.01) were also strongly negatively correlated with death anxiety. The analyses supported their roles as independent mediators (effect sizes: self-efficacy pathway = -0.1203; resilience pathway = -0.0647). Furthermore, a chain mediation model showed that the sequential pathway through “self-efficacy → psychological resilience” was significant (effect size = -0.1373), accounting for 31.29% of the total indirect effect. Collectively, the three mediation paths explained 73.46% of the total association. Notably, psychological resilience was the strongest negative correlate of death anxiety (β = -0.463).

**Conclusion:**

Our cross-sectional findings suggest that physical exercise is not only directly associated with lower death anxiety in older adults, but may also be linked through a chain of psychological resources involving self-efficacy and psychological resilience. These findings offer a theoretical basis for future longitudinal and interventional research aimed at constructing integrated “exercise-psychology” intervention models.

## 1 Introduction

According to the United Nations World Social Report 2023, the global population aged 65 and above reached 761 million in 2021, and this figure is projected to increase to 1.6 billion by 2050. The World Health Organization (WHO) Global Strategy on Aging and Health (2016) highlights that the primary disease burden in later life stems from non-communicable diseases (NCDs), rendering the risk factors for chronic diseases and mental disorders critical targets for health promotion initiatives. With advancing age, older adults develop heightened awareness of mortality, making death anxiety a significant psychological concern affecting this population.

Death anxiety is a common psychological response to the awareness of life’s end (1), and in recent years it has drawn increasing attention due to its complex associations with mental health, quality of life, and disease prognosis. Studies have shown that death anxiety is significantly positively correlated with psychological disorders such as depression and anxiety (2), and it may exacerbate physical symptoms in patients with chronic diseases by influencing health-related decision-making and behaviors (3). Clinically, cancer patients often exhibit particularly high levels of death anxiety due to their increased exposure to disease progression and mortality threats (4), which dynamically interacts with variables such as psychological flexibility and perceived self-burden (5). However, existing research predominantly focuses on specific disease populations or single intervention strategies, lacking systematic integration of multidimensional mechanisms and cross-group comparisons (6). For instance, healthcare workers face unique challenges regarding death anxiety due to the nature of their profession, yet the moderating effects of variables such as compassion fatigue and professional identity remain unclear (7).

Moreover, the COVID-19 pandemic has led to a widespread increase in death anxiety and has had long-term impacts on social mental health (8), highlighting the urgent need to investigate buffering mechanisms under crisis conditions (9). Notably, psychological interventions such as Acceptance and Commitment Therapy (ACT) and CALM therapy have demonstrated potential in alleviating death anxiety (10), yet the heterogeneity of their underlying mechanisms warrants further investigation (11). As a result, integrating multidisciplinary perspectives and constructing dynamic models encompassing biological, psychological, and social factors has become a pivotal direction in current death anxiety research. This study aims not only to optimize clinical intervention strategies but also to provide theoretical support for public health policymaking.

## 2 Literature review and research hypotheses

### 2.1 Physical exercise and death anxiety in older adults

In recent years, the relationship between physical exercise and death anxiety in older adults has gradually become a focal point in the fields of psychology and geriatric medicine. Especially with the COVID-19 pandemic causing a large number of people to be infected worldwide (12), it has increased the anxiety of death among the elderly. Numerous studies have demonstrated that death anxiety, as a pervasive psychological phenomenon, is closely related to individuals’ mental health, social support, and coping strategies (1, 13). In older populations, death anxiety is often exacerbated by chronic illnesses, cognitive decline, and changes in social roles (14). Against this backdrop, physical exercise—a modifiable lifestyle intervention—has been shown to significantly reduce death anxiety levels among the elderly through various multidimensional mechanisms, including enhanced physiological functioning and increased social interaction (15). For example, Srivastava and Ghosh (16) noted that regular physical activity can indirectly alleviate fear and unease about death by releasing endorphins, improving sleep quality, and enhancing self-efficacy. Furthermore, a cross-sectional study by Li et al. ^8^ found that elderly individuals who engaged in long-term physical exercise scored significantly lower on the CT-DAS (Death Anxiety Scale) compared to their sedentary counterparts, with this relationship remaining robust even after controlling for gender, education level, and chronic disease status.

Additional research indicates that the inhibitory effect of physical exercise on death anxiety may be achieved through an enhancement of life meaning and psychological flexibility. In an intervention experiment based on Acceptance and Commitment Therapy (ACT), Chen et al. (2024) found that physical exercise helped older adults reconstruct their perceptions of life value, thereby reducing negative associations with death (17). Similarly, Yan et al. (2024) verified through structural equation modeling that physical exercise indirectly reduced death anxiety by strengthening family cohesion and social support networks (18). It is noteworthy that the effect of physical exercise on death anxiety exhibits a dose-response characteristic. For instance, a longitudinal study by Husain et al. (19) (2024) demonstrated that older adults who engaged in moderate-to-vigorous exercise for more than 150 minutes per week exhibited approximately a 23.6% reduction in death anxiety compared to those with insufficient exercise. This finding is corroborated by neurophysiological evidence, which suggests that regular exercise can modulate the functional connectivity between the prefrontal cortex and amygdala, thereby reducing the sensitivity to death-related threat perceptions ^4^. Based on the aforementioned evidence, this study proposes the following hypothesis:

H1: Physical exercise has a negative effect on death anxiety in older adults, meaning that greater levels of physical exercise are associated with lower death anxiety.

### 2.2 The mediating role of self-efficacy

The alleviating effect of physical exercise on death anxiety in older adults has garnered increasing attention, and self-efficacy—individuals’ subjective evaluation of their own capabilities—may play a key mediating role in this relationship. Research has shown that death anxiety is significantly associated with health deterioration, social isolation, and a diminished sense of life meaning among the elderly (20), while regular physical exercise can effectively mitigate these risk factors by enhancing physiological function and psychological resilience (21, 22). According to Bandura’s (1997) social cognitive theory, physical exercise reinforces individuals’ confidence in their own abilities through the successful achievement of exercise-related goals, thereby increasing their sense of control over life events—a mechanism that is particularly pronounced in older populations (23). A longitudinal study by Guo et al. demonstrated that older adults engaging in moderate-intensity exercise weekly exhibited a significantly greater improvement in self-efficacy scores compared to non-exercisers, with the enhancement in self-efficacy accounting for 41% of the effect of physical exercise on reducing death anxiety (24).

The mediating role of self-efficacy may be realized through multiple pathways. First, physical exercise directly improves bodily functions, thereby bolstering older adults’ confidence in managing their health and reducing death-related fears caused by physical decline (25, 26). Second, group exercise provides positive social feedback through interpersonal interactions, which in turn fosters the accumulation of self-efficacy and buffers the exacerbating effect of loneliness on death anxiety (27). Additionally, neuroimaging evidence suggests that long-term exercise can enhance the functional connectivity between the prefrontal cortex and hippocampus, thereby improving executive function and emotional regulation, both of which are positively related to self-efficacy^11^. For instance, an fMRI study involving participants over 65 years old found that a 6-month aerobic exercise intervention significantly increased self-efficacy, which was associated with enhanced inhibition of the default mode network (DMN), and these neuroplastic changes predicted the degree of reduction in death anxiety (28).

Based on the evidence presented, the following hypotheses are proposed:

H2: Physical exercise has a positive effect on self-efficacy.
H3: Self-efficacy has a negative effect on death anxiety in older adults.
H4: Self-efficacy mediates the relationship between physical exercise and death anxiety in older adults.

### 2.3 The mediating role of psychological resilience

Physical exercise is recognized as an effective intervention for reducing death anxiety in older adults, and psychological resilience—a core psychological resource for coping with adversity—may play a critical mediating role in this process. Research indicates that death anxiety is significantly associated with declines in physiological functioning, a lack of social support, and reduced sense of meaning in life among the elderly (29, 30). Regular physical exercise can systematically enhance psychological resilience by activating the neuroendocrine system and promoting social participation (31). According to the stress-buffering theory, psychological resilience aids older adults in reconstructing their cognitive appraisal of death-related threats by improving emotional regulation and cognitive flexibility, thereby reducing anxiety responses (32).

The mediating pathway of psychological resilience may operate through several mechanisms. First, physical exercise can improve the functional connectivity between the prefrontal cortex and the limbic system, enhancing executive control and emotional regulation, which in turn increases individuals’ capacity to cope with death-related stimuli^11^. Neuroimaging evidence has shown that a 6-month aerobic exercise intervention reduced amygdala activation by 32% when elderly participants were exposed to death-related cues, and this reduction was significantly negatively correlated with improvements in psychological resilience scores (33). Second, group-based physical exercise can strengthen psychological resilience through enhanced social identification and a sense of belonging, thereby buffering the impact of loneliness on the exacerbation of death anxiety (34). It is also important to note that the mediating effect of psychological resilience may be moderated by baseline health status; among older adults with chronic diseases, the positive impact of physical exercise on psychological resilience is more pronounced, with the mediating pathway explaining up to 61% of the effect (35). On the basis of these findings, the following hypotheses are proposed:

H5: Physical exercise has a positive effect on psychological resilience.
H6: Psychological resilience has a negative effect on death anxiety in older adults.
H7: Psychological resilience mediates the relationship between physical exercise and death anxiety in older adults.

### 2.4 The chain-mediating role of psychological resilience and self-efficacy

The mechanism by which physical exercise alleviates death anxiety in older adults has gradually shifted from a single mediating pathway to the exploration of multidimensional chain models. In this context, the synergistic effects of psychological resilience and self-efficacy may form a critical mediating chain. Research indicates that death anxiety is significantly associated with declines in physiological function, the loss of social roles, and a diminished sense of life meaning among older adults (36, 37). Regular physical exercise, by activating neuroplasticity and reinforcing behavioral mechanisms, can systematically enhance self-efficacy and bolster psychological resilience (32, 38). According to the integrated theory of social cognition and stress buffering, physical exercise initially strengthens individuals’ beliefs in their own capabilities (i.e., self-efficacy) through the successful attainment of exercise goals. This, in turn, promotes the accumulation of psychological resources (i.e., psychological resilience) to better adapt to stress, ultimately reducing the over-alertness to death-related threats (39).

The biological basis of this chain-mediating pathway can be further elucidated through neuroendocrine and brain network mechanisms. Functional near-infrared spectroscopy (fNIRS) studies have shown that physical exercise enhances the functional connectivity of the prefrontal-limbic system, thereby improving executive control. Increases in self-efficacy are positively correlated with activation intensity in the dorsolateral prefrontal cortex (DLPFC), while enhancements in psychological resilience are negatively correlated with the functional coupling between the amygdala and anterior cingulate cortex (ACC). These findings suggest that self-efficacy, as an initial mediator, optimizes cognitive resource allocation and provides a neural foundation for the regulation of emotional responses by psychological resilience (26). Based on these findings, the following hypotheses are proposed:

H8: Psychological resilience positively influences self-efficacy.
H9: Psychological resilience and self-efficacy jointly play a chain-mediating role in the relationship between physical exercise and death anxiety in older adults.

**Figure 1:**
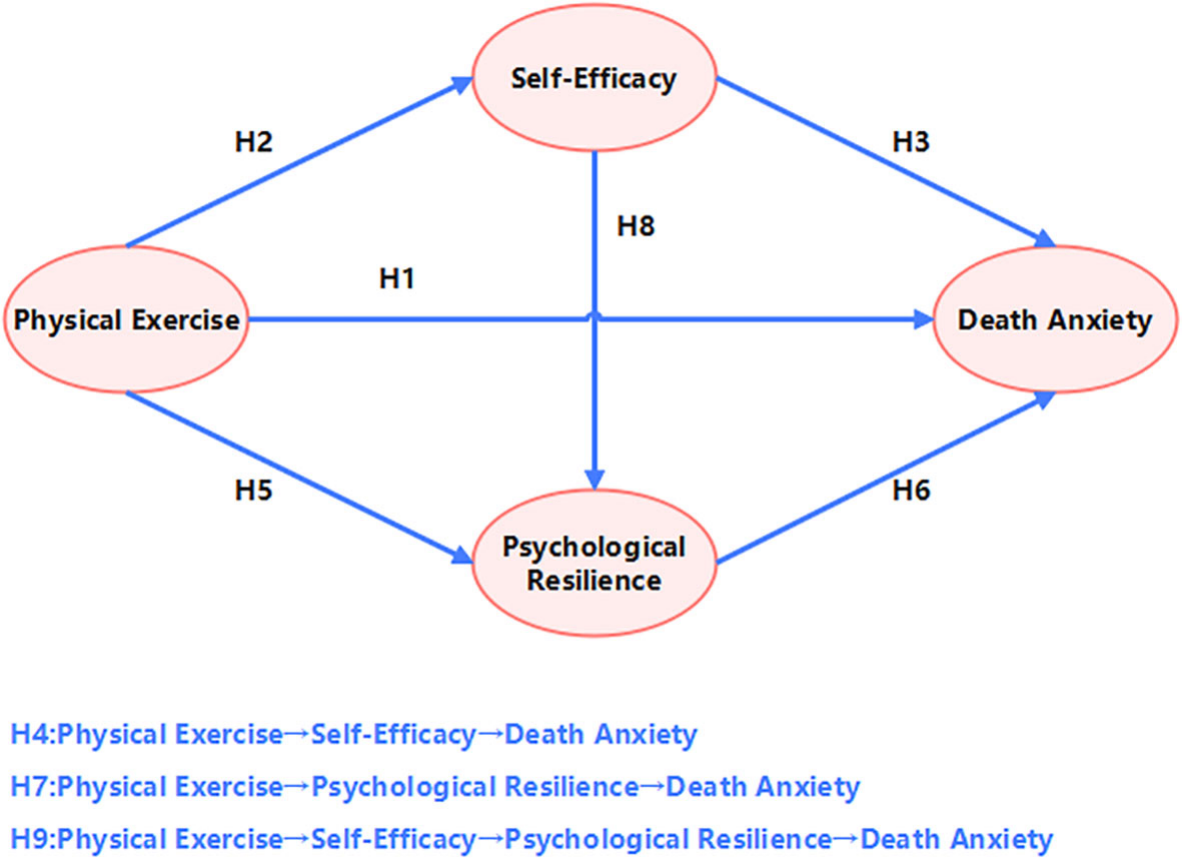
Hypothetical model.

The research hypotheses are shown in **Figure 1**.

## 3 Research methods

### 3.1 Participants

This study strictly adheres to the World Health Organization’s age definition for elderly populations in developing countries, targeting individuals aged 60 and above. The sample’s age distribution is divided into four groups: 60–64 years (32.6%), 65–69 years (28.2%), 70–74 years (25.4%), and 75–80 years (13.7%), ensuring representation across multiple subgroups within the elderly population. This study was conducted in accordance with local legislation and institutional requirements, and participants provided written informed consent to participate in this study. A total of 800 questionnaires were distributed, and 772 valid responses were received, yielding a high response rate of 96.5%, which indicates good data quality. Exclusion criteria for the questionnaires included participants under 60 years, incomplete responses, patterned responses, unable to exercise due to illness or disability, or logical inconsistencies; a total of 28 invalid questionnaires were excluded. The gender distribution in the sample was balanced, with males accounting for 51.2% and females for 48.8%, thereby effectively reducing the risk of gender bias. Geographical distribution included the eastern (34.6%), central (34.6%), and western (30.8%) regions, with nearly equal representation from rural (50.1%) and urban (49.9%) areas, demonstrating a certain degree of representativeness in terms of location and urban–rural dimensions. Additionally, 57% of the participants reported no history of chronic disease, whereas 43% had a chronic condition (see **Table 1**).

**Table 1:** Distribution of demographic variables of survey participants.

Variable	Category	Frequency	Percent	Valid percent
Age	60~64	252	32.6	32.6
	65~69	218	28.2	28.2
	70~74	196	25.4	25.4
	75~80	106	13.7	13.7
Gender	Male	395	51.2	51.2
	Female	377	48.8	48.8
Region	Eastern	267	34.6	34.6
	Central	267	34.6	34.6
	Western	238	30.8	30.8
Residence	Rural	387	50.1	50.1
	Urban	385	49.9	49.9
Chronic Disease	No	440	57	57
	Yes	332	43	43

### 3.2 Research instruments

#### 3.2.1 Physical Exercise Rating Scale

The Physical Activity Rating Scale (PARS-3), revised by Liang Deqing, was used to assess participants’ levels of physical exercise (40). This scale evaluates exercise amount based on three dimensions: intensity, frequency, and duration, using a 5-point Likert scoring system. Exercise amount is calculated with the formula “Exercise Amount = Exercise Frequency × (Exercise Duration - 1) × Exercise Intensity,” yielding a score range from 0 to 100. Based on the total score, exercise levels are classified into three categories: low (≤19 points), moderate (20–42 points), and high (43–100 points), serving as the evaluation indicator for participants’ physical activity. In this study, the scale yielded a KMO value of 0.705 and a Bartlett’s test value of 681.892 (p < 0.001), indicating good structural validity. The questionnaire’s Cronbach’s α coefficient was 0.790, demonstrating acceptable reliability.

#### 3.2.2 Death Anxiety Scale

This study employed the Death Anxiety Scale developed by Professor Templer in 1967, which has been widely applied in research on older adults after localization (41). The scale is unidimensional and comprises 15 items scored on a 5-point scale ranging from 1 (strongly disagree) to 5 (strongly agree). Nine items are positively scored, while the remaining six items are reverse-scored. In the context of Chinese culture, the scale exhibits good psychometric properties (42). Higher scores on the Death Anxiety Scale indicate greater levels of death anxiety. In this study, the scale produced a KMO value of 0.942 and a Bartlett’s test value of 2354.200 (p < 0.001), demonstrating sound structural validity. The Cronbach’s α coefficient was 0.851, indicating good reliability.

#### 3.2.3 Self-Efficacy Scale

Self-efficacy was measured using the General Self-Efficacy Scale (43), which includes 10 items. Each item is scored from 1 (“not at all true”) to 4 (“exactly true”), yielding a total score range from 10 to 40, with higher scores reflecting stronger self-efficacy. In this study, the scale demonstrated a KMO value of 0.904 and a Bartlett’s test value of 1439.719 (p < 0.001), indicating good structural validity. The Cronbach’s α coefficient was 0.803, reflecting acceptable reliability.

#### 3.2.4 Psychological Resilience Scale

The Resilience Scale was originally developed by Smith et al. (44) to measure psychological resilience among college students. This study employed the Brief Resilience Scale, which was refined by Campbell-Sill et al. from the original 25-item CD-RIS to form a more concise and practical unidimensional scale consisting of 10 core items. After careful translation and cultural adaptation by domestic scholars, the scale has been successfully applied to elderly populations and has been validated in multiple studies as a reliable and effective tool for measuring psychological resilience in older adults (45). In this study, to reduce the response burden on elderly participants and to better align with local characteristics, four items were removed. The final version of the scale consists of 6 items, among which items 2, 4, and 6 are reverse-scored. Responses were collected using a Likert 5-point scale. The scale demonstrated good structural validity in this study, with a KMO value of 0.810 and a Bartlett’s sphericity test value of 695.704 (p < 0.001). The Cronbach’s α coefficient for the questionnaire was 0.721, indicating good reliability.

### 3.3 Data processing and analysis

The data were statistically analyzed using SPSS 27.0 and PROCESS 4.0 software. The main steps included:

1. Classifying, converting, and computing the valid data;
2. Employing Harman’s single-factor test to examine common method bias;
3. Assessing the reliability and structural validity of the measurement instruments via Cronbach’s α coefficients and Bartlett’s test values;
4. Using correlation analysis and linear regression to verify the relationships among the variables and to assess whether physical exercise, psychological resilience, and self-efficacy significantly influence death anxiety in older adults;
5. Applying the Bootstrap method to test the mediating effects of psychological resilience and self-efficacy, both as independent mediators and as a chain mediation in the relationship between physical exercise and death anxiety.

## 4 Research results

### 4.1 Test for common method bias

Common method bias was assessed using statistical techniques. Through Harman’s single-factor test, an exploratory factor analysis (EFA) was conducted on all measurement items. The first unrotated factor explained 33.71% of the total variance, which is below the critical threshold of 40%, indicating that there is no severe common method bias in the data. Although the single factor did not explain the majority of the variance, further measures were implemented during questionnaire design to control potential bias, including:

1. 1 Ensuring anonymous responses;
2. 2 Separating the order of items related to independent and dependent variables;
3. 3 Utilizing multiple-source scales and ensuring clarity in item wording.

Subsequent analyses may also incorporate techniques such as controlling for unmeasured latent method factors or introducing marker variables for additional validation. Overall, the data quality meets the basic requirements for statistical analysis.

### 4.2 Correlation analysis of research variables

Descriptive statistics and correlation analyses for the relevant variables are presented in **Table 2**. The means and standard deviations indicate that the average score for physical exercise among the surveyed older adults was 1.31 (SD = 1.10), suggesting overall low levels of physical activity with considerable individual variation. The average death anxiety score was 2.98 (SD = 0.65), which is at a moderately high level. The mean scores for self-efficacy (M = 2.84, SD = 0.62) and psychological resilience (M = 3.08, SD = 0.78) were close to the theoretical midpoints, although individual differences in psychological resilience were relatively more pronounced.

**Table 2:** Mean, standard deviation, and correlation coefficients of research variables.

Variable	M	SD	Physical exercise	Death anxiety	Psychological resilience	Self-efficacy
Physical Exercise	1.31	1.10				
Death Anxiety	2.98	0.65	-0.740**			
Self-Efficacy	2.84	0.62	0.714**	-0.827**		
Psychological Resilience	3.08	0.78	0.736**	-0.854**	0.869**	

**p<0.01.

Correlation analyses revealed that physical exercise was highly and significantly negatively correlated with death anxiety (r = -0.740, p < 0.01), suggesting that increased frequency or intensity of exercise is associated with lower levels of death anxiety in older adults. Additionally, physical exercise was significantly positively correlated with self-efficacy (r = 0.714, p < 0.01) and psychological resilience (r = 0.736, p < 0.01), indicating that exercise may promote the accumulation of internal psychological resources. The strong negative correlations between death anxiety and both self-efficacy (r = -0.827, p < 0.01) and psychological resilience (r = -0.854, p < 0.01) further support the potential mediating roles of these variables in alleviating death anxiety. Notably, self-efficacy and psychological resilience exhibited an extremely strong positive correlation (r = 0.869, p < 0.01), suggesting that these two factors may operate synergistically, which is consistent with the assumptions of the chain mediation model.

This study further explored the independent predictive effects of physical exercise, self-efficacy, and psychological resilience on death anxiety in older adults through separate regression analyses, with results presented in **Table 3**. All three regression models reached high levels of significance. In the model predicting death anxiety from physical exercise, the unstandardized regression coefficient was -0.44 (SE = 0.01, β = -0.74, p < 0.001), indicating that for each one-unit increase in physical exercise, the level of death anxiety decreased by 0.44 units, and that physical exercise alone accounted for 55% of the variance in death anxiety (R² = 0.55). The regression coefficient for self-efficacy was -0.86 (SE = 0.02, β = -0.83, p < 0.001), with an even stronger explanatory power (R² = 0.68), suggesting that for every one-unit increase in self-efficacy, death anxiety decreased by 0.86 units; its standardized coefficient indicates that the effect of self-efficacy on death anxiety is slightly higher than that of physical exercise. The model with psychological resilience demonstrated the most significant predictive effect, with a regression coefficient of -0.71 (SE = 0.02, β = -0.85, p < 0.001) and an explained variance of 73% (R² = 0.73), indicating that psychological resilience exerts the strongest negative prediction on death anxiety among the three variables.

**Table 3:** Independent regression analysis of physical exercise, self-efficacy, and psychological resilience on death anxiety in the elderly.

Variable	Elderly death anxiety						
	B	SE	β	T	F	R^2^	R^2adj^
Constant	3.56	0.03					
Physical Exercise	-0.44	0.01	-0.74	-30.51	930.97	0.55	0.55
Constant	5.43	0.06					
Self-Efficacy	-0.86	0.02	-0.83	-40.74	1659.99	0.68	0.68
Constant	5.17	0.05					
Psychological Resilience	-0.71	0.02	-0.85	-45.61	2080.56	0.73	0.73

Regarding model fit, the F-values for the three independent variables were 930.97, 1659.99, and 2080.56 respectively (all p < 0.001), demonstrating the overall high statistical power of the models. A comparison of the standardized regression coefficients (β) further revealed the relative strengths of the variables’ effects: the effect size of psychological resilience (β = -0.85) was slightly higher than that of self-efficacy (β = -0.83), while physical exercise (β = -0.74) had a somewhat weaker influence. This result is consistent with the trends observed in the inter-variable correlation coefficients in **Table 2**, particularly the strong negative correlation between psychological resilience and death anxiety (r = -0.854), which is quantitatively confirmed here. Moreover, the adjusted R² values for all three models were nearly identical to the original R² values, indicating that the models did not suffer from overfitting due to an increased number of variables.

This study explored the joint predictive effects of physical exercise, self-efficacy, and psychological resilience on death anxiety in the elderly using multiple regression analysis, with results as shown in **Table 4**. The model indicates that all three independent variables significantly and negatively predict death anxiety. Specifically, the unstandardized regression coefficient for physical exercise was -0.116 (SE = 0.015, β = -0.196, p < 0.001), indicating that, when controlling for other variables, each one-unit increase in physical exercise is associated with a 0.116-unit decrease in death anxiety; however, its standardized coefficient suggests that its independent explanatory power is relatively weak. The regression coefficient for self-efficacy was -0.296 (SE = 0.037, β = -0.284, p < 0.001), implying that for every one-unit increase in self-efficacy, death anxiety decreases by 0.296 units, and its standardized effect size is slightly higher than that of physical exercise. The predictive effect of psychological resilience is the most pronounced, with a regression coefficient of -0.385 (SE = 0.030, β = -0.463, p < 0.001), indicating that each unit increase in psychological resilience is associated with a 0.385-unit reduction in death anxiety; its standardized coefficient (β = -0.463) is significantly higher than those of the other variables, further validating the central role of psychological resilience in alleviating death anxiety.

**Table 4:** Multiple regression analysis of physical exercise, self-efficacy, and psychological resilience on death anxiety in the elderly.

Variable	Elderly death anxiety					
	B	SE	β	t	Tolerance	VIF
Constant	5.162	0.062		82.785		
Physical Exercise	-0.116	0.015	-0.196	-7.587	0.436	2.293
Self-Efficacy	-0.296	0.037	-0.284	-8.026	0.233	4.288
Psychological Resilience	-0.385	0.030	-0.463	-12.657	0.218	4.585

The multicollinearity diagnostics of the model revealed that the tolerance values for physical exercise, self-efficacy, and psychological resilience were 0.436, 0.233, and 0.218, respectively, and the corresponding variance inflation factors (VIF) were 2.293, 4.288, and 4.585. The VIF values of all variables exceeding 5 indicate that there is no obvious collinearity among the variables. This finding is consistent with the high correlation (r = 0.869) observed between them in **Table 2**.

### 4.3 Mediation effect testing

This study employed the bootstrap method to test the chain-mediating effects of self-efficacy and psychological resilience in the relationship between physical exercise and death anxiety, with results presented in **Table 5**. To assess the alignment between the theoretical model and the empirical data, we examined multiple fit indices. The results indicated an excellent model fit: χ²/df = 1.151, CFI = 0.956, RMSEA = 0.014, SRMR = 0.031. All indices exceeded their respective thresholds for good fit (χ²/df < 3, CFI > 0.95, RMSEA < 0.05, SRMR < 0.05), demonstrating that the model constructed in this study fits the data exceptionally well, thereby justifying subsequent path coefficient analysis.

**Table 5:** Results of mediation effect path testing.

Effect	Path	Effect size	SE	LLCL	ULCL	Effect ratio (100%)
Total Effect	Direct Path	-0.4387	0.0144	-0.4670	-0.4105	100.00
Direct Effect		-0.1164	0.0153	-0.1466	-0.0863	26.53
Total Indirect Effect		-0.3223	0.0163	-0.3574	-0.2928	73.46
Indirect Effect	Path 1	-0.1203	0.0158	-0.1528	-0.0913	27.42
	Path 2	-0.0647	0.0091	-0.0836	-0.0478	14.75
	Path 3	-0.1373	0.0131	-0.1636	-0.1131	31.29

The total effect of the model was -0.4387 (SE = 0.0144), indicating that each one-unit increase in physical exercise was associated with an overall reduction of 0.4387 units in death anxiety. Of this total effect, the direct effect was -0.1164 (SE = 0.0153), accounting for 26.53% of the total effect, while the total indirect effect was -0.3223 (SE = 0.0163), representing 73.46% of the overall effect. This finding suggests that the combined mediating pathway of self-efficacy and psychological resilience is the primary mechanism by which physical exercise alleviates death anxiety.

More specifically, mediation pathway analysis revealed the following:

Path 1 (Physical Exercise → Self-Efficacy → Death Anxiety) had an effect size of -0.1203 (95% CI: -0.1528 to -0.0913), accounting for 27.42% of the total indirect effect.
Path 2 (Physical Exercise → Psychological Resilience → Death Anxiety) had an effect size of -0.0647 (95% CI: -0.0836 to -0.0478), accounting for 14.75%.
Path 3 (Physical Exercise → Self-Efficacy → Psychological Resilience → Death Anxiety) exhibited the most substantial chain-mediating effect, with an effect size of -0.1373 (95% CI: -0.1636 to -0.1131), accounting for 31.29% of the total indirect effect.

**Figure 2:**
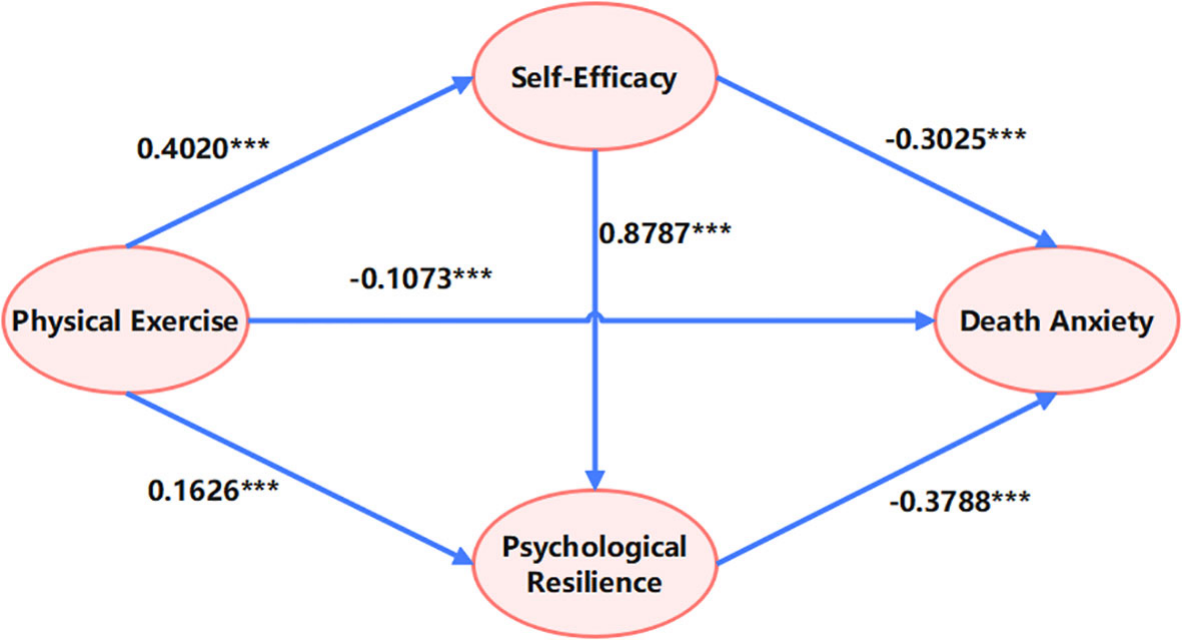
Chain mediation model of self-efficacy and psychological resilience. ***p<0.001.

None of the confidence intervals for these pathways included zero, which confirms the statistical significance of the mediation effects (see **Figure 2**).

**Figure 3:**
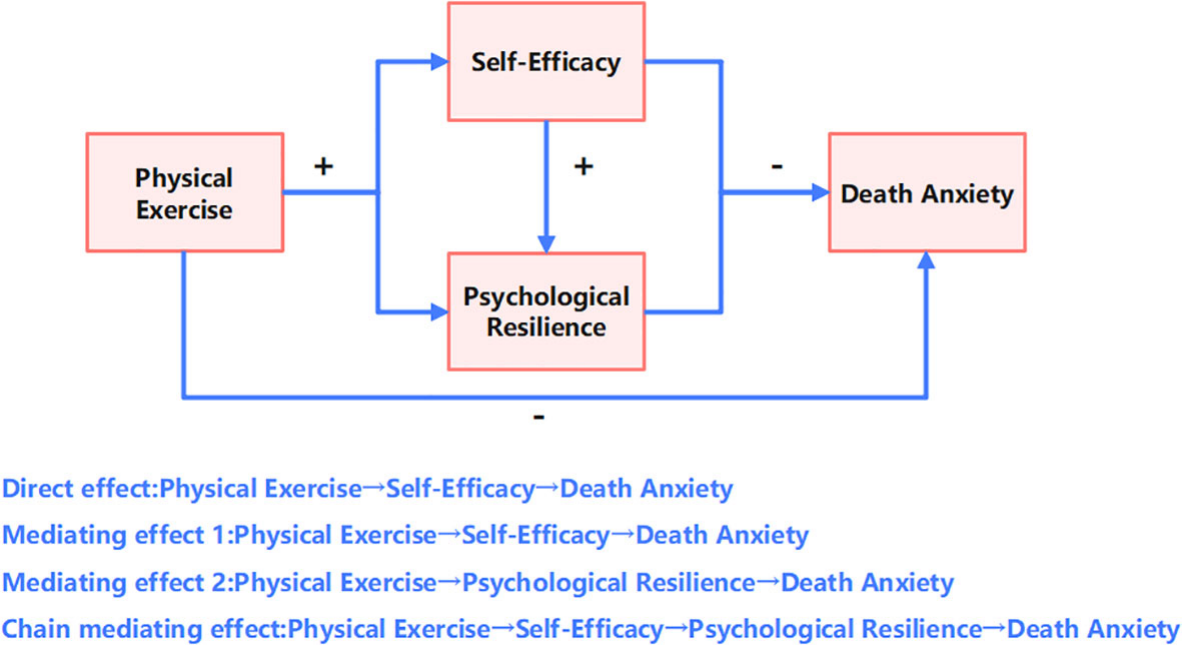
Path model.

The prominent contribution of the chain mediation pathway (Path 3) indicates that self-efficacy not only directly alleviates death anxiety but also exerts an additional indirect protective effect by enhancing psychological resilience. This finding is consistent with the hierarchical accumulation of psychological resources as posited by Conservation of Resources theory. The combined contribution of Path 1 and Path 3 (58.71%) is significantly higher than that of Path 2, highlighting the pivotal role of self-efficacy in the model (see **Figure 3**). This may be attributed to self-efficacy’s direct influence on individual behavioral control and cognitive appraisal, which more readily facilitates subsequent improvements in psychological resilience. Notably, although psychological resilience demonstrated the strongest predictive power in the separate regression analyses (see **Table 5**), its independent mediation pathway (Path 2) showed a relatively limited effect size. This suggests that the impact of psychological resilience is more dependent on the activation of self-efficacy rather than operating independently.

## 5 Discussion

### 5.1 The association between physical exercise and death anxiety in the elderly

The mechanisms by which physical exercise influences death anxiety in the elderly can be interpreted from multiple theoretical dimensions. Previous research has demonstrated that death anxiety is not only closely related to an individual’s psychological state but is also significantly moderated by social support and cognitive restructuring abilities^1^. In the context of exercise interventions, physical exercise may exert its effects through the enhancement of self-efficacy as a mediator, a mechanism that is inherently associated with improvements in psychological flexibility^5^.

In elderly populations, the social dimension of physical exercise warrants special attention. Group-based exercise activities create a sense of collective identity that effectively compensates for the existential crises often stemming from the loss of social roles in later life. This compensatory mechanism is analogous to the cultural worldview defense proposed in terror management theory, wherein the subculture formed by exercise groups may serve as a new buffering system against death anxiety. Longitudinal studies have shown that mind–body exercises such as Tai Chi have a unique effect on promoting reflection on death; this cognitive restructuring ability can transform death anxiety into a sense of life meaning^15^. Compared with purely cognitive behavioral therapies, exercise interventions have a distinct advantage in improving body image concerns, which is particularly valuable for alleviating the prevalent anxieties related to physical aging among the elderly.

However, existing research still presents some theoretical gaps. First, the dose-response relationship between exercise intensity and the alleviation of death anxiety remains unclear; overly intense exercise might exacerbate anxiety due to physical discomfort. Second, the differential effects of various types of exercise require further delineation, as differences between group exercise and individual training in terms of social support may impact intervention outcomes (46). Furthermore, the moderating role of cultural factors on the effectiveness of exercise interventions deserves deeper exploration; for instance, traditional Eastern practices that embody specific views on life and death may offer unique psychological protective mechanisms. Future research should aim to establish interdisciplinary integrative models that associate exercise physiology indicators with psychological measures, while also investigating the long-term effects of sustained exercise habits on acceptance of death during end-of-life care stages (47). Such theoretical breakthroughs would not only refine the framework of healthy aging but also provide scientific evidence for designing targeted exercise intervention programs.

### 5.2 The mediating role of psychological resilience

Psychological resilience plays a multi-layered psychosocial mediating role in the effect of physical exercise on death anxiety in the elderly. Previous studies have indicated that physical exercise, by enhancing physiological functions and social engagement ^47^, indirectly improves an individual’s capacity to cope with stress and adversity, with psychological resilience—as a core indicator of dynamic adaptability—potentially serving as a crucial mediator in this process (48). From the perspective of the socioemotional selectivity theory, the heightened awareness of the finiteness of life in the elderly makes them more susceptible to death anxiety (49). Yet, regular physical activity, by activating neuroplasticity and cognitive reappraisal functions, may facilitate the development of psychological resilience, thereby reconstructing the individual’s cognitive framework regarding death threats^15^. It is important to note that psychological resilience is reflected not only in emotional regulation but also in the restructuring of one’s meaning system (50). For example, physical exercise, through the enhancement of self-efficacy and goal-directed behavior^9^, enables the elderly to transform death anxiety into a quest for life meaning. This transformation mechanism is closely related to the consolidation of a secure base as proposed in attachment theory, where individuals with high psychological resilience are more likely to buffer the negative effects of death anxiety via social support networks.

### 5.3 The mediating role of self-efficacy

Self-efficacy also plays a mediating role in the relationship between physical exercise and death anxiety among the elderly, reflecting multi-dimensional psychological adaptation mechanisms. Existing research indicates that death anxiety is significantly positively correlated with psychological distress^1^, and as a core capability for coping with stress (51), self-efficacy may weaken the negative impact of death threats by restructuring an individual’s cognitive appraisal system. For instance, death education has been shown to alleviate anxiety by enhancing the sense of life meaning (52), which aligns with the pathway by which self-efficacy promotes cognitive reappraisal. As an exogenous intervention, physical exercise may indirectly increase levels of self-efficacy—through improvements in bodily functioning and the enhancement of social support networks (e.g., by strengthening family cohesion)—thus forming a “physiological–psychological–social” chain protective effect. Notably, research on psychological interventions among cancer patients suggests that enhancing psychological flexibility through the acceptance of death can reduce anxiety^5^, implying that physical exercise might bolster self-efficacy via similar acceptance mechanisms.

### 5.4 Chain-mediating role of psychological resilience and self-efficacy

This study examined the mediating role of psychological resilience in the relationship between physical exercise and death anxiety in the elderly, with the results indicating that psychological resilience significantly buffers the negative impact of death anxiety by enhancing individuals’ emotional regulation and cognitive restructuring abilities (53). Previous findings have shown that physical exercise, through the activation of neuroplasticity and the promotion of social connection ^47^, can indirectly elevate levels of psychological resilience, which is consistent with the findings of this study. From the perspective of socioemotional selectivity theory, the heightened perception of life’s finitude in the elderly increases their susceptibility to death anxiety; however, physical exercise, by strengthening self-efficacy, may help individuals reconstruct their perception of death threats (54). It is important to note that the role of psychological resilience is not confined solely to emotional management but also involves the integration of one’s meaning system. For example, processes such as life review or spiritual reflection, which have been observed to enhance the sense of meaning in interventions involving advance care planning among terminal patients (55), echo similar mechanisms in which exercise interventions may bolster psychological resilience.

### 5.5 Community-based mental health practice program for older adults​

Against the backdrop of accelerated population aging, the design of community health programs needs to transcend traditional single-dimensional physiological health interventions and instead establish an integrated “exercise-psychology-social” collaborative service system. For instance, communities could leverage existing public fitness facilities to develop intervention programs centered on group-based physical activities such as Tai Chi, square dancing, or walking groups. Regular collective activities not only enhance older adults’ physical functioning but also strengthen their social belongingness and sense of self-control. This multidimensional empowerment mechanism directly increases exercise participation while simultaneously fostering self-efficacy in health management through sustained positive feedback loops within group interactions, thereby laying the foundation for developing psychological resilience.

At the practical level, community health services should emphasize stepwise cultivation of psychological resources. Given the significant proportion of chain-mediated effects, phased psychological support modules should be embedded within exercise interventions. In the initial phase, self-efficacy can be strengthened through micro-goal setting and immediate achievement feedback during early-stage physical exercise sessions. Subsequently, stress-coping skill training combined with post-exercise group sharing sessions helps older adults transfer exercise-derived mastery experiences into cognitive restructuring of life meaning. Finally, cross-generational interaction initiatives such as “Exercise Mentor Programs” can be implemented, where senior residents serve as community volunteers guiding youth sports activities. This dual mechanism of role empowerment and social contribution further consolidates psychological resilience through enhanced social identity and purpose-driven engagement.

## 6 Conclusion and outlook

### 6.1 Conclusion

This study proposed a model elucidating the complex interrelationships between physical exercise, self-efficacy, psychological resilience, and death anxiety in older adults. The findings suggest that physical exercise is not only directly associated with lower death anxiety but may also be linked to it through the mediating roles of internal psychological resources. As a core psychological variable, self-efficacy was both directly associated with lower death anxiety and, by virtue of its strong positive correlation with psychological resilience, appears to form a significant chain-mediating pathway that plays a dominant role in the overall model. Although psychological resilience demonstrated the strongest independent statistical association with death anxiety, its role within the model is synergistic with self-efficacy, reflecting a hypothesized hierarchy in the accumulation of psychological resources. The strong correlations among the variables indicate that self-efficacy and psychological resilience may function in a complementary and synergistic manner, potentially forming a psychological protective network against death anxiety. The study lends support to the idea that the potential benefits of physical exercise for mitigating death anxiety may not operate in isolation but could be realized through the integration of multi-layered psychological resources. Methodologically, the use of multi-stage bias control and statistical tests ensured the robustness of the observed associations, providing reliable evidence for the proposed model. Overall, this study systematically outlines a set of plausible pathways influencing death anxiety in the elderly and emphasizes the pivotal role of psychological factors, thereby providing a theoretical foundation for future longitudinal and experimental research into the links between health behaviors and psychological states.

### 6.2 Limitations

This study has several limitations. First, although the cross-sectional design can reveal associations among variables, it cannot establish causality. Future research should employ longitudinal tracking or experimental interventions to verify the temporal effects of physical exercise on death anxiety. Second, although the sample covers both urban and rural areas and different regions, it did not include elderly individuals who are unable to participate in physical exercise due to health restrictions, which may lead to selection bias. Third, self-reported data may be influenced by social desirability bias, and the tendency of the elderly to avoid discussing death might weaken the validity of the measurements. Although measures such as anonymous responses were adopted, future studies should consider incorporating physiological indicators or multiple data sources to enhance ecological validity. Fourth, while the chain-mediating model was statistically validated, it did not control for potential confounding variables such as religious beliefs and economic status, which may affect the purity of the mediation pathways. Fifth, the Physical activity assessment scale is very short and may not fully capture the multi-dimensional aspects of the exercise habits of the elderly. Finally, since this study is based on the Chinese cultural context, caution is needed when generalizing the conclusions to other cultural settings. Future research should conduct cross-cultural comparisons to test the universality of the model.

### 6.3 Future outlook

Future research can further expand both the theoretical and practical boundaries by deeply integrating the needs of an aging society with advancements in intelligent technologies. As global aging accelerates, the mental health issues of the elderly have become a core public health governance challenge, necessitating the development of an “active health” intervention system to address the complexity and prevalence of death anxiety. Methodologically, future studies may rely on artificial intelligence to develop multimodal data integration models that combine physiological indicators from wearable devices (e.g., heart rate variability, exercise trajectories), linguistic sentiment features analyzed via natural language processing (NLP), and neuroelectrophysiological signals captured by brain-computer interfaces (BCI) to construct dynamic risk prediction systems. Such systems could accurately identify high-risk groups for death anxiety and enable early warning. Additionally, incorporating digital twin technology to simulate the dynamic effects of various exercise interventions on individual psychological states in a virtual space, and employing reinforcement learning to optimize personalized intervention parameters, holds great promise.

For special elderly populations such as those who are disabled or live alone, innovative immersive metaverse rehabilitation platforms could be developed. These platforms would use virtual reality (VR) and augmented reality (AR) technologies to reconstruct exercise scenarios, overcoming physical limitations by allowing bedridden elders to engage in “compensatory exercise” through neuro-muscular electrical stimulation paired with virtual avatars. This can be combined with AI-driven emotional companionship robots to deliver cognitive-behavioral interventions, forming an integrated digital intervention network that addresses physiological, psychological, and social dimensions.

In terms of mechanism exploration, it is essential to deepen interdisciplinary research at the intersection of brain science and digital pathology. Applying deep learning to multi-omics data (such as genomic, epigenomic, and microbiome data) to elucidate the association networks with psychological resilience phenotypes could reveal the cross-scale biological mechanisms underlying the effects of physical exercise on death anxiety. Concurrently, developing blockchain-based elderly health databases to enable secure data sharing across institutions and regions would provide a data foundation for constructing a national-level mental health ecosystem for the elderly.

On the practical translation front, efforts should be made to promote the development of “smart healthy aging communities.” This could involve integrating AI-driven personalized exercise prescription systems into primary healthcare, achieving a coordinated response between home, community, and hospitals via 5G-enabled IoT technologies, and developing digital literacy courses tailored to the elderly to bridge the “silver digital divide.” Furthermore, attention must be paid to the cultural reconstruction of death perceptions in the post-pandemic era. Utilizing generative AI to create localized digital content for death education, combined with targeted dissemination through social media algorithms, may help reshape a positive view of aging. Ultimately, through multidimensional innovation driven by technology, data, and cultural integration, we can shift the paradigm of death anxiety intervention from “generalized” to “precise,” and from “passive coping” to “proactive prevention.”

## Data Availability

The original contributions presented in the study are included in the article/Supplementary Material. Further inquiries can be directed to the corresponding author.
